# Decoding the Lipid Droplet Proteome: New Frontiers in Cardiovascular Disease Research

**DOI:** 10.3390/ijms262110280

**Published:** 2025-10-22

**Authors:** Alice Mallia, Giulia G. Papaianni, Lisa Brocca, Cristina Banfi, Erica Gianazza

**Affiliations:** Centro Cardiologico Monzino IRCCS, Unit of Functional Proteomics, Metabolomics and Network Analysis, Via Parea 4, 20138 Milan, Italy; alice.mallia@cardiologicomonzino.it (A.M.); giulia.papaianni@cardiologicomonzino.it (G.G.P.); lisa.brocca@cardiologicomonzino.it (L.B.); cristina.banfi@cardiologicomonzino.it (C.B.)

**Keywords:** lipid droplets, proteomics, omics approaches, cardiovascular diseases

## Abstract

Lipid droplets (LDs) are cellular dynamic organelles involved in lipid storage and maintaining lipid balance. They contain many proteins on their surface that significantly affect their functions and behaviors. Thus, it becomes crucial to characterize the LD proteome using appropriate “omics” technologies that may contribute to an accurate understanding of the LD roles in human diseases. Indeed, LDs and their associated proteins are involved in several pathologies related to dysfunctional lipid metabolism, such as hyperlipidemia, obesity, metabolic syndrome, and cardiovascular diseases (CVDs). This review aims to provide an overview of “omics” studies focused on characterizing the LD proteome in the cardiovascular field, offering additional insight into the involvement of LDs in the development and progression of heart complications, as well as defining potential targets useful for diagnostic, preventive, and therapeutic approaches for patients.

## 1. Lipid Droplets: Structure and Functions

Lipid droplets (LDs) are cellular dynamic organelles with a diameter that can reach up to 100 μm, encircled by a 2–2.5 nm single phospholipid monolayer addressed with different proteins and devoted to storing neutral lipids in the hydrophilic cytosol. Several functions of LDs are common between bacteria and humans, such as lipid storage and metabolism [1]. LDs regulate lipid metabolism and protect against lipotoxicity and oxidative damage by sequestering excess fatty acids (FAs) and preventing the accumulation of reactive lipid species. Emerging evidence also suggests that LDs can bind nucleic acids, providing a site where protein–nucleic acid complexes can associate to modulate gene expression or protect RNAs during stress or infection [2]. Moreover, LDs can be involved in viral replication and assembly [3,4].

The first remark of cellular LDs goes back to the last decade of the nineteenth century, but more than a century was necessary to first characterize these organelles [5].

As mentioned before, their function is not only limited to lipid storage, but they are involved in many aspects of cellular physiology [6]. LDs are important in cellular metabolism, and they are cyclically generated and degraded based on cell demand. The storage of neutral lipids is a reservoir for energy generation or membrane synthesis, and the biogenesis of LDs is also crucial in the protection from lipotoxicity induced by the accumulation of FAs, toxic glycerolipids, and sterols in cell membranes [7]. LD biogenesis takes place in the endoplasmic reticulum (ER) through coordinated lipid synthesis and budding mechanisms, but LD degradation also occurs through tightly controlled pathways like cytosolic lipolysis and autophagy-dependent lipophagy. The modulation of these processes is influenced by metabolic status, stress signals, and nutrient availability [8].

## 2. Lipid Droplet Biogenesis and Proteins Involved

**Figure 1:**
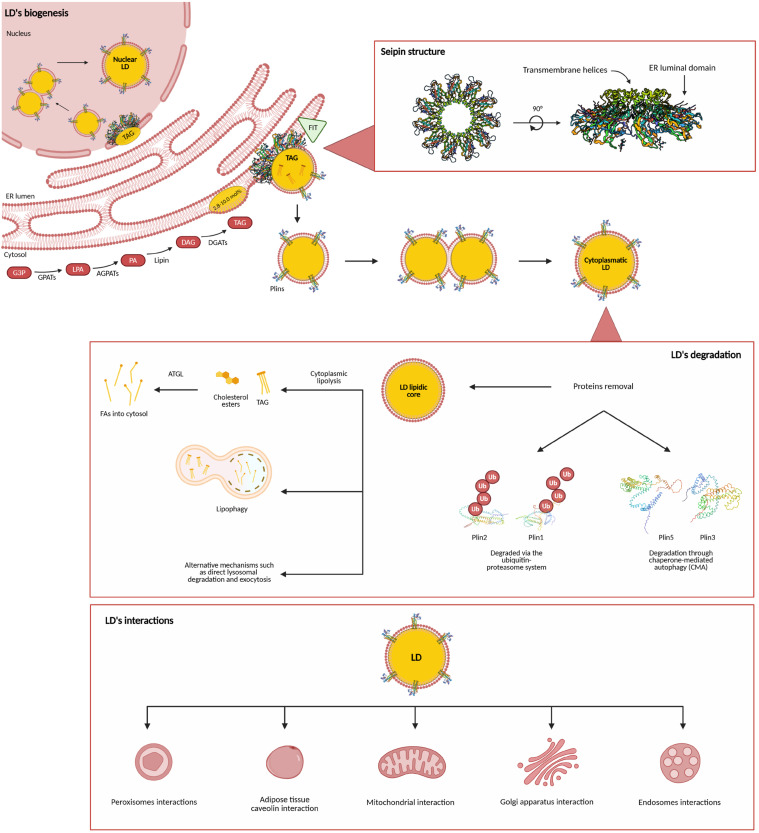
Schematic view of the LD biogenesis process with a focus on the proteins involved. In particular, the structure of the seipin protein is shown. The mechanism of LD degradation is also shown, with a focus on the fate of the LD’s surface proteins and the remaining lipid core. The main interactions of the LDs with cellular compartments are also reported. AGPAT, acyl-G3P-acyltransferase; ATGL, adipose triglyceride lipase; DAG, diacylglycerol; DGAT, diacylglycerol O-acyltransferase; ER, endoplasmic reticulum; FIT, fat storage-inducing transmembrane protein; G3P, glycerol-3-phosphate; GPAT, glycerol-3-phosphate acyltransferase; LD, lipid droplet; LPA, lysophosphatidic acid; PA, phosphatidic acid; Plins, perilipins; TAG, triacylglycerols; Ub, ubiquitin. Created in Biorender. L.B. (2025) https://app.biorender.com (accessed on 20 October 2025).

The biophysical properties of the ER membrane influence the LDs assembly site. Indeed, in vitro, in vivo, and in silico modeling studies demonstrated that phospholipid composition and membrane tension are crucial for LD generation [9,10]. The asymmetric distribution of membrane phospholipids in the ER at LD nascent sites influences the membrane tension and curvature, affecting the direction of emersion of the budding organelle [11]. Phospholipids with negative intrinsic curvature, such as diacylglycerols (DAGs), favor the embedding of the LDs within the ER bilayer, whereas lipids with positive charge reduce the surface tension, determining the budding of LDs from the ER [12]. Moreover, it has been recently demonstrated that the architecture of the ER also has a role in the nucleation of LDs. Indeed, organelle biogenesis is more prominent in ER tubules with respect to ER sheets because seipin, which is a key enzyme in LDs budding and ER-LD contact site maintenance, preferentially locates in tubule ER subdomains [13,14]. The multiple steps involved in the biogenesis of LDs are summarized in Figure 1.

### 2.1. Cytoplasmic Lipid Droplets

Cytoplasmic LDs are by far the best characterized among the different organelle subclasses. Their biogenesis was deeply investigated, and various models and steps for LD formation were hypothesized [15,16,17,18].

The most supported version involves the synthesis, nucleation, cytoplasmic budding, and growth of neutral lipids from the ER [11]. Newly synthesized neutral lipids, including sterol esters and triacylglycerols (TAGs), deposit into the ER membrane and freely diffuse between the phospholipid acyl chains within the hydrophobic phase of the ER membrane [19]. As they accumulate over the critical concentration of 2.8–10.0 mol%, a lens is formed via the demixing phenomenon of phase separation [19]. The lens formation can occur spontaneously or be influenced by membrane tension, curvature, or local lipid and protein composition [13,20]. This phenomenon is induced when the interaction of neutral lipids among themselves becomes thermodynamically more favorable than their interaction with membrane components [21].

In an emerging model, it was hypothesized that TAG aggregation and phase separation, even at low TAG concentrations, are promoted by the interaction of TAGs with the hydrophobic transmembrane segments and the hydrophobic helix in the oligomeric cage-like seipin ring complex [22]. This phenomenon facilitates the formation of LDs at specific sites of the ER, preventing spontaneous generation at random sites [23]. The lipid lens keeps on growing, accumulating lipids, until a sphere is postulated and wrapped by the cytosolic monolayer of the ER, finally budding the nascent LD in the cytoplasm.

Biophysically, LDs are oil-in-water emulsion droplets that are expected to undergo spontaneous destabilization processes [24]. The thermodynamic instability of droplets is due to the unfavorable interface between the immiscible fluids, which generates surface tension. To reduce the interface, droplets tend to mix their contents [25]. It can happen in two different ways: fusion of LDs, a rapid merging of or coalescence of droplets, or Ostwald ripening, a process by which smaller droplets transfer material to bigger ones through a connecting phase [21].

Several enzymes are crucial in the LD biogenesis process. The enzymes responsible for the production of neutral lipids that compose the core of LDs are essential for initial LD biogenesis. Among them, glycerol-3-phosphate acyltransferase (GPAT), acyl-G3P-acyltransferase (AGPAT), and diacylglycerol O-acyltransferase (DGAT) 1 and 2 regulate at different levels LD biosynthesis and growth, catalyzing neutral lipid synthesis [26]. GPAT and AGPAT enzymes are involved in the generation of DAGs, precursors of TAGs, through the sequential acetylation of glycerol-3-phosphate. Further, the lipin enzymes (lipin 1–3) are key regulators of phospholipids and TAG biosynthesis, and consequently, of LDs enlargement [27]. DGAT1 enzyme is mainly expressed in the ER lumen, whereas DGAT2 is active toward the cytosol side of the ER membrane. TAGs produced on both sides of ER are included in nascent LDs [28]. Moreover, enzymes devoted to phospholipid synthesis, such as phosphocholine cytidylyltransferase (CCT), play a crucial role in LD generation [29]. In concert with phospho- and neutral lipid synthesis enzymes, other proteins are fundamental in LDs biogenesis, including fat storage-inducing transmembrane (FIT) proteins, seipin, perilipins (Plins), and proteins that modulate ER shaping [30].

FIT proteins are an evolutionarily conserved family of enzymes that facilitate the budding of LDs; in mammals, there are two different isoforms, FIT1 and FIT2 [31]. They are localized on the ER membrane and have a high affinity for TAGs and DAGs. FIT1 is mainly expressed in oxidative tissues like skeletal muscle and heart, while FIT2 has a wider tissue expression, with the highest levels in adipose tissue. The specific mechanism by which these proteins promote LD budding is still unclear, but it is evident that they are necessary for the correct budding of droplets from the ER because they improve LD nucleation by sequestering TAGs at specific locations on the ER surface [32]. FIT2 is involved in one of the steps of LDs biogenesis, possibly promoting local DAG removal and allowing the progression from lens-like to maturely budded [10]. Its depletion results in the accumulation of neutral lipids and nascent LDs that fail to bud towards the cytosol, remaining partially exposed to the ER lumen. Thus, this protein could be involved in the restoration of the cytosolic layer of ER during directional LD budding [32].

Seipin is a crucial enzyme in the biogenesis and homology of LDs because it renders ER subdomains permissive for LD biogenesis (Figure 1). It is a transmembrane enzyme composed of two transmembrane helices positioned in proximity and a large ER luminal domain [12]. Recent studies provided new important insights into the structural model, assembly, and mechanisms of action of seipin [33,34,35,36], revealing that it assembles into a decameric, cage-like complex at LD formation sites within the ER. Using cryo-electron microscopy combined with artificial intelligence (AI)-guided modeling, Arlt et al. showed that seipin lumenal domains form a stable ring, while its transmembrane segments adopt two alternating conformations facilitated by flexible switch regions critical for LD biogenesis [33]. Functional analyses demonstrated that while the lumenal domain alone is sufficient for oligomerization, the transmembrane segments are essential for maintaining complex stability and supporting proper LD formation. The study proposes a model in which a closed seipin cage promotes TAG phase separation, subsequently adopting an open conformation to enable LD growth and budding. Despite the critical role of seipin in LD biogenesis, the deficiency of this protein is not associated with the abrogation of organelle budding but results in the dysregulation of LD biogenesis and growth, with the generation of giant LDs [37]. Seipin forms a functional complex with LDAF1 (also known as Promethin), which associates with expanding LDs and is thought to lower the TAG concentration threshold necessary for LD nucleation, while seipin itself facilitates phase transitions of TAGs within the ER bilayer [38]. As a scaffold protein, seipin recruits essential lipid metabolic enzymes such as lipin 1 and AGPAT2, thereby regulating phosphatidic acid (PA) metabolism at ER-LD junctions. It also modulates the activity of GPAT3 and GPAT4, key enzymes in de novo lipid synthesis, and interacts with FIT proteins, which are necessary for LD budding from the ER. Additionally, its interaction with 14-3-3β and cofilin-1 suggests a role in actin cytoskeleton remodeling and adipogenesis [39]. Structurally, seipin contains a β-sandwich luminal domain capable of binding anionic phospholipids and features a ring-shaped oligomeric configuration that can trap TAGs and their precursor DAG. Simulations and imaging studies propose that seipin localizes to lipid-packing defects within the ER, concentrating at nascent “oil lens” sites, where it promotes localized lipid remodeling and facilitates directional TAG transfer into emerging LDs [40]. The seipin complex also establishes a diffusion barrier at ER-LD contact points, preserving local lipid composition and preventing disruptions to overall ER morphology. Dysfunctional seipin, resulting from mutations in the *BSCL2* gene, causes congenital generalized lipodystrophy type 2, a severe inherited disorder characterized by near-complete loss of adipose tissue, likely due to impaired mesenchymal stem cell differentiation into preadipocytes [41].

### 2.2. Nuclear Lipid Droplets

In addition to conventional LDs residing in the cytosol, recent studies have also identified the existence of nuclear lipid droplets (nLDs), a distinct subpopulation localized within the nucleus. This class of LDs was initially considered as cytoplasmic LDs entrapped in the nucleus, but they are now emerging as organelles with their distinct characteristics. Depending on the cell type where they reside, different biogenesis mechanisms of nLDs have been identified. In hepatocytes, nLDs arise from lipoprotein precursors formed in the ER lumen. During very-low-density lipoprotein (VLDL) assembly, the microsomal triglyceride transfer protein mediates the generation of ApoB-containing particles and ApoB-free lumenal lipid droplets; under conditions of ER stress, when ApoB synthesis is reduced, the ApoB-free precursors can instead give rise to nLDs that accumulate within the nucleus. In non-hepatocyte cells, nLDs originate from *de novo* generation at the inner nuclear membrane, or from the internalization of luminal LDs via nucleoplasmic reticulum, the dynamic network of branched membrane invaginations extending from the nuclear envelope into the nucleus. The biogenesis of nLDs appears to occur at the inner nuclear membrane (INM), where both lipid biosynthetic enzymes and lipases are present, supporting the notion that the nuclear environment possesses the machinery necessary for local nLD formation and turnover [42]. This process resembles cytoplasmic LD biogenesis at the ER but occurs in a topologically distinct compartment. TAG synthesis at the INM generates a lens-like accumulation of neutral lipids between the bilayer leaflets, which subsequently buds toward the nucleoplasm. The site of this budding appears to be defined by seipin and its associated proteins, though evidence suggests that seipin-independent mechanisms may also operate. The membrane invaginations of the nuclear envelope may facilitate this process by providing regions of positive curvature that favor droplet nucleation [43]. Seipin exerts a nuanced and tightly regulated role in the biogenesis and control of nLDs, as highlighted in recent structural and functional studies [44,45]. Although its canonical localization is at ER–LD contact sites, evidence indicates that seipin can influence lipid homeostasis at the INM. Seipin has been detected at or near the INM, where it may assemble into ring-like oligomers that locally concentrate TAG and DAG, facilitating the nucleation of nLDs. The hydrophobic helices within the luminal domain of seipin are proposed to interact with neutral lipids to stabilize nascent TAG lenses and coordinate the curvature of the INM during droplet emergence [46]. Seipin deficiency leads to an aberrant increase in nLD number and PA accumulation within the nuclear envelope, suggesting that seipin primarily acts from the ER to restrain neutral lipid deposition at the INM. This is thought to occur through its role in buffering DAG and PA levels, thereby maintaining lipid homeostasis and preventing ectopic TAG nucleation inside the nucleus. In this context, seipin indirectly safeguards nuclear membrane integrity and chromatin organization by regulating lipid flux between the ER and the nuclear envelope [19]. Thus, seipin’s function in nLD biology appears to be bimodal: under certain stress or lipid-loading conditions, it may facilitate the controlled formation of nLDs by organizing lipid microdomains at the INM; under normal conditions, it acts as a negative regulator, preventing spontaneous TG lens formation through lipid composition control. Overall, these findings position seipin as a central orchestrator of nuclear–ER lipid communication, integrating structural, metabolic, and spatial mechanisms that determine when and where lipid droplets can form within the nuclear compartment. While some reports suggest that nLDs may arise from lipoprotein precursors, others propose a seipin-independent mechanism originating directly from the INM, highlighting ongoing uncertainties regarding their precise biogenetic pathway [47]. Indeed, it has been observed that seipin is not directly involved in nLD formation because it is not detected at the INM, but it indirectly influences their biogenesis by regulating PA levels and lipin 1 expression. In addition, knockdown of seipin leads to an increase in nLDs and connections with the INM [48]. Functionally, nLDs are closely associated with nuclear lipid metabolism; the localized conversion of PA to TAG within the INM can alter the distribution of PA and DAG, facilitating the formation and growth of nLDs and promoting TAG accumulation within them. The accumulation of nLDs and the resultant lipid microdomains within the INM may influence nuclear membrane fluidity, lateral protein mobility, and clustering, potentially impacting nuclear functions proximal to histones and gene promoters [49].

The accumulation of nLDs within the nucleus causes cellular dysfunction and disease, leading to abnormal nuclear structure, altered gene expression, and impairment of DNA repair mechanisms [50]. It has been observed that the abnormal growth of nLDs is closely associated with modifications in the nuclear lamina protein LMN-1 [51], confirming that the nuclear envelope components and LD functionality are linked.

This progressive and marked nuclear lipid deposition is a typical characteristic of aging, a multifactorial process that collectively contributes to a gradual and irreversible decline of cellular functions, leading to cellular senescence, systemic decline, and age-related diseases. In particular, the accumulation of LDs contributes to the pathogenesis of various neurodegenerative diseases, including Alzheimer’s disease, Parkinson’s disease, and Huntington’s disease, with mechanisms that still need to be clarified. It has recently been observed that a strong buildup of LDs occurs in neurons, microglia, myeloid cells, astrocytes, and ependymal cells in the elderly brain, promoting the aging process (i.e., ER stress, lipotoxicity, and mitochondrial damage) and accelerating disease progression at advanced stages [52,53,54]. Conte et al. suggested that only Plin2 is modulated with age in the brain, and the abnormal expression of LDs is a hallmark of inflammation and neurodegeneration [55]. Similarly, DGAT2 is another protein involved in the microglial LD formation and is increased in Alzheimer’s disease brains [56].

The nuclear lipid balance is controlled and maintained by the adipose triglyceride lipase (ATGL)-1, which is localized in both the cytoplasm and the nuclear envelope. This enzyme has been identified as a potential therapeutic target for reducing nLD accumulation and enhancing nuclear health in age-related disorders [50]. Caloric restriction and reduced insulin signaling, for example, can increase ATGL-1 activity, thus preserving nuclear function. Furthermore, changes in nuclear lipid homeostasis have been linked to metabolic disorders, such as fatty liver disease and obesity, which suggests that similar pathways may be important not only for aging but also for disease pathology.

Steatosis involves LDs accumulating in liver cells, both in the cytoplasm and in the nucleus. The specific role of nLDs is still unclear. Still, it has been demonstrated that they are frequently observed in pathological conditions like non-alcoholic steatohepatitis [57], in which the formation of cytoplasmic LDs in nucleoplasmic reticulum is inhibited, thus suggesting that nLDs do not directly reflect the cytoplasmic lipid accumulation. In addition, nLDs are involved in gene expression regulation and phosphatidylcholine synthesis during periods of ER stress [58].

Instead, nLD depletion can be influenced by several factors, including changes in cell growth and metabolism, and dysregulation of specific proteins that control LD biogenesis and turnover. The depletion of proteins like torsinA or LAP1 can cause an aberrant deposition of lipids in the nucleus following the block of their mobilization to the cellular sites, such as the mitochondria for cellular energy generation by β-oxidation or the secretion pathway through the VLDL secretion [59]. In addition, the activation of nuclear-specific lipases during the nuclear expansion in late oogenesis can also lead to the removal of lipids from nLDs [51].

Nuclear and cytoplasmic LDs differ in origin, localization, and functions [43]. Cytoplasmic LDs are more abundant and found in the cytoplasm, connected with various organelles, while nLDs are fewer in number and located in the nucleus. Nuclear LDs are separated from the cytoplasmic organelles, thus giving them unique functional characteristics.

The origin of these two types of LDs is also different because cytoplasmic LDs form in the ER, while nLDs form following exposure to excess fatty acids or during cellular stress.

Collectively, while cytoplasmic LDs are primarily involved in energy storage, proteostasis, and stress responses, nLDs represent a specialized nuclear lipid storage system with unique biogenetic origins and potential roles in lipid metabolism, nuclear organization, and signaling. Of course, it is crucial to investigate the proteomic and lipidomic profiles of nLD to better understand their important roles, but it is a challenge to isolate sufficient amounts of nLDs without any contamination by cytoplasmic LDs. In a recent study, for the first time, rat-liver-nLD proteome was evaluated under physiological and nonpathological conditions [60] after nLD isolation and protein concentration before the GeLC-MS^2^. The identified proteins were involved in several functions, including cytoskeleton structure, transcription and translation, histones, protein-folding and PTM, cellular proliferation, lipid metabolism, and transport. Among them, it has been confirmed the expression of carboxylesterase 1d in purified nLDs, which is an important enzyme from the lipid–metabolism pathway.

In the future, a better knowledge of the nLD roles in the pathophysiology of diseases could also improve the therapy of several diseases through the targeted manipulation of nLDs.

However, even though interventions targeted at nLDs have promising outcomes, several questions and problem issues remain open. The components of these organelles need to be characterized using a multi-omic approach, and their detection optimized for precise live monitoring. Indeed, traditional biochemical approaches have been applied to profile the composition of extracted LDs, but these approaches lose spatial information regarding their distribution and heterogeneity. In recent years, advanced techniques have allowed for in-depth and spatially resolved detection of nLDs in vivo, but they still require elaborate sample preparation and data interpretation. The visualization and quantification of nLDs in living cells with high spatial and temporal resolution is possible through fluorescent probes or genetically encoded reporters. Non-invasive label-free techniques, such as coherent Raman scattering (CARS/SRS) microscopy, have also been developed to allow high chemical specificity and spatiotemporal resolution for quantifying LDs without the use of exogenous labels, which can interfere with cellular function. However, these in vivo imaging methods have the limitation of often being non- or semi-quantitative, and do not have the specificity to distinguish LDs with different compositions [61].

## 3. Lipid Droplet Degradation and Associated Proteins

LDs are not merely passive lipid storage organelles, but dynamic structures actively degraded to release FAs for energy production and other cellular processes. Several major degradation pathways have been identified, including cytoplasmic lipolysis and lipophagy, alongside alternative mechanisms such as direct lysosomal degradation and exocytosis [62].

Cytoplasmic lipolysis, one of the principal routes, involves the action of cytosolic lipases that hydrolyze neutral lipids, primarily triacylglycerols and cholesterol esters, stored within the LD core, thereby releasing free FAs into the cytosol [63]. The key enzyme mediating this process is ATGL, whose activity is modulated by co-factors such as ABHD5 (also known as CGI-58). Members of the perilipin family, particularly Plin1 and Plin5, regulate lipolysis by controlling the access of lipases to the LD surface. Plin1 interacts with ABHD5, while Plin5 not only facilitates ATGL activity but also mediates the metabolic connection between LDs and mitochondria, supporting FA transfer for β-oxidation [64].

In parallel, lipophagy constitutes a second major degradation pathway, whereby LDs are delivered to lysosomes for hydrolysis by acid lipases. Lipophagy may occur via macrolipophagy, which employs canonical autophagic machinery to sequester LDs in autophagosomes that subsequently fuse with lysosomes, or through microlipophagy, which involves the direct engulfment of LDs at membrane contact sites with lysosomes [65]. FAs released through this route may exit lysosomes via fusion with the plasma membrane (lysosomal exocytosis), allowing cells to regulate intracellular lipid levels [66].

Both Plin1 and Plin2 can be degraded via the ubiquitin–proteasome system, particularly during lipid mobilization, while other LD-associated proteins, like Plin3 and Plin5, are also subject to selective degradation through chaperone-mediated autophagy (CMA), facilitating LD breakdown [67]. Indeed, the removal of these proteins by CMA is crucial for allowing cytosolic lipases like ATGL and macroautophagy proteins to access the LD lipid core and break down lipids, as well as promote LD degradation via lipophagy.

Overall, LD degradation is governed by tightly coordinated pathways involving lipases, regulatory proteins (e.g., Plins and ABHD5), the autophagic–lysosomal system, and alternative mechanisms such as exocytosis. These pathways exhibit functional crosstalk and cooperation, although several mechanistic details remain to be elucidated [68].

## 4. Lipid Droplet Interaction with Other Organelles

LDs form extensive membrane contact sites with various cellular compartments, as demonstrated by multispectral imaging studies that reveal temporally coordinated interactions critical for lipid homeostasis, metabolite exchange, and inter-organelle communication [69]. LDs closely interact with cellular compartments through membrane contact sites, facilitating the exchange of lipids and proteins and coordinating essential metabolic functions (Figure 1) [70].

One of the most well-characterized interactions occurs between LDs and the ER, from which LDs originate. These contacts, mediated by proteins such as seipin, are crucial for LD biogenesis and expansion.

LDs also establish functional connections with mitochondria, forming the so-called peridroplet mitochondria (PDMs), which enable the transfer of FAs for β-oxidation and ATP production [71]. Notably, PDMs exhibit distinct bioenergetic properties, cristae architecture, and proteomic profiles compared to typical cytoplasmic mitochondria (CMs), with PDMs favoring pyruvate oxidation to support TAG synthesis and CMs utilizing free FAs via β-oxidation. This mitochondrial interaction is regulated in part by Plin family proteins, particularly Plin5 [72]. The LD-associated protein Plin5 establishes both physical and metabolic connections with mitochondria, interacting with fatty acid transport protein 4 at contact sites to promote FAs transfer [73]. Rab8a has also been identified as a potential mitochondrial receptor for LDs in skeletal muscle [74]. Moreover, a functional metabolic circuit connecting LDs, peroxisomes, and mitochondria has been proposed to regulate energy expenditure in adipose tissue [75].

Peroxisomes exhibit stable interactions with LDs, particularly in yeast, where they can form protrusions known as “pexopodia” that invade the lipid core of LDs. These structures may facilitate hemifusion events, enabling the direct transfer of FAs for peroxisomal β-oxidation, thereby contributing to lipid detoxification and energy metabolism [76]. Proteins such as spastin and peroxisomal biogenesis factor 5 (PEX5) coordinate LD-peroxisome crosstalk, which may influence processes such as lipolysis during fasting and lifespan extension through the metabolism of ether lipids and monounsaturated FAs [77].

Beyond these organelles, LDs interact with components of the Golgi apparatus, including the COPI/COPII vesicular trafficking machinery, ER-Golgi intermediate compartment, and trans-Golgi network, where they may be involved in lipid transport via vacuolar protein sorting-associated protein 13B-mediated transfer [78].

LDs also contact early and late endosomes, with Rab proteins regulating these interactions, and contribute to lipophagy via lysosomes or vacuoles [79].

In adipocytes, LDs interface with caveolae, with cholesterol-induced caveolin trafficking supporting caveolar endocytosis [80].

Collectively, these multifaceted interactions, mediated by specialized proteins, underscore the central role of LDs in coordinating lipid metabolism, inter-organelle communication, and cellular homeostasis far beyond their traditional function as inert lipid reservoirs. The molecular mediators of these contacts represent a growing focus in cell biology and metabolic research to understand how they regulate lipid transfer, energy metabolism, and cellular health, with implications for various diseases [81].

Additionally, the localization of vesicular trafficking proteins on LD surfaces, including SNAREs and Rab GTPases, suggests that molecular machinery involved in vesicle transport, docking, and fusion may also function at LD membranes, potentially facilitating inter-organelle communication and the regulated exchange of lipids and proteins [82].

## 5. Lipid Droplet-Associated Proteins Classification and Characterization

LDs in different tissue and cell types have unique characteristics, such as specific size, lipid composition, and regulatory mechanisms, as well as distinct LD proteins [83]. Therefore, LDs present a remarkable heterogeneity both in protein and lipid composition. Their structural differences reflect the organelles’ different populations with peculiar functions [84].

LDs are mainly characterized by their associated proteins, which are able to significantly affect their functions and behaviors. The LD proteome includes many lipid metabolic enzymes, regulatory scaffold proteins, proteins involved in LD clustering and fusion, membrane-trafficking proteins, and additional proteins with still unknown functions [85]. Proteins target LDs from the ER and cytoplasm [86].

LD proteins are divided into two categories: class I and class II proteins. Class I proteins are primarily linked to the ER membrane and can move into LDs, but can be found in the ER even in the absence of LDs. Despite the membranes of ER and LDs being continuous, the proteomes of these organelles remain unique and the mechanisms that determine the protein trafficking between them are strictly controlled. The membrane junction acts as a semi-permeable protein diffusion barrier that regulates the trafficking of specific proteins [87]. They are able to target LDs from the ER bilayer through a hydrophobic hairpin structure. The targeting regions are composed by an α-helical domain that forms a v-shaped sequence that inserts in the membrane; this structural modification is induced by the presence of a proline residue in the middle of the hydrophobic sequence that causes the protein fold [88]. In addition to ER proteins with internal hairpins, other class I proteins have N-terminal hydrophobic sequences that are necessary and sufficient to target both the ER and LDs. The structure of these N-terminal LD targeting domains is not known [88]. Some class I proteins localize in LDs at later time points by trafficking across a distinct membrane bridge formed by a set of fusion machinery which includes SNARE proteins [22]. Class I proteins lack a luminal domain; this characteristic allows them to easily insert into the ER bilayer or the LD monolayer, and to move from one to the other during LD formation or after budding via membrane bridges [88]. What causes class I proteins to accumulate on LDs is still unclear. Class I proteins that go from the ER to LDs through membrane bridges could probably balance out between the ER and LD compartments. Nevertheless, a large number of proteins, including GPAT4, build up on LD surfaces in far greater quantities than the ER pool [89]. The mechanism by which class I LD proteins are removed from LDs is unknown. Because class I proteins contain highly hydrophobic LD binding motifs likely interacting with the LD core, they probably require dedicated machinery to extract them from membranes. This could occur either after relocalization of the protein to the ER, where ER-associated degradation (ERAD) removes the protein, or by direct extraction and degradation at the LD [90]. Although this process is still unclear, both the relocalization and turnover of class I LD proteins appear to be regulated by lipid-loading state. Some class I proteins might be degraded directly on the surface of LDs [67]. Several proteins involved in protein degradation localize to LDs and could mediate such a process [91]. Class I proteins include GPAT4, DGAT2, and ACSL3. Class II proteins are synthetized in cytosolic ribosomes and translated from the cytosol to the LD surface thanks to amphipathic helices or short hydrophobic-rich sequences. How class II proteins distinguish LD surfaces from other membranes, such as the ER bilayer, is unknown. Not all proteins with amphipathic helices, for example, appear to prefer binding to LDs [68]. The binding of class II proteins to LD surfaces is likely influenced by factors other than lipid composition. For example, conical lipids that induce packing defects in membranes, such as DAG, promote CCT binding to membranes, similarly to other amphipathic helices [92]. Class II proteins engage with the phospholipid monolayer through several processes, among which are lipid anchors, amphipathic helices, and protein–protein interactions [93]. Displacement by macromolecular crowding appears to be a major mechanism for removal of some class II proteins. Under conditions that encourage class II protein targeting to LDs, the combination of amphipathic helix folding to surface binding results in a very stable binding reaction. However, this pathway is not just inverted when these proteins dissociate from LDs. Rather, proteins unfold after falling off LDs. Furthermore, binding is probably practically irreversible under steady-state conditions due to the dimerization of several LD proteins or the concatenation of multiple binding helices. However, as LDs shrink during lipolysis, their surface compresses, which likely leads to macromolecular crowding of LD proteins due to the increasing protein density at the surface [94]. Among class II proteins, Plins and CCT can be identified [95]. The main characteristics of the proteins belonging to these classes are described in Table 1.

The most studied LD structural proteins are probably the Plin proteins [96], but many other molecules still need further investigation.

LD-associated proteins are structurally or enzymatically involved in LD homeostasis, and their functions and molecular networks have been extensively studied in the last few years. The tasks of LDs are strictly related to the composition of the LD proteome, whose characterization is crucial for an accurate understanding of the LD roles in human diseases. Indeed, LDs and their associated proteins are involved in several pathologies associated with dysfunctional lipid metabolism, such as cardiovascular diseases (CVDs) and cardiometabolic disorders [97].

Therefore, thoroughly defining the LD proteome, the functions, and the mechanisms of regulation of the LD proteins is critical to determining the central role of LDs in health and disease.

The most important challenges in understanding the pathophysiological roles of LDs are the optimization of protocols for isolating LDs from a wide range of tissues and characterizing their lipid content and associated proteins using multidisciplinary approaches, as well as generating databases for LD proteins and lipids [98] that can help users to discover key molecules in human metabolism.

**Figure 2:**
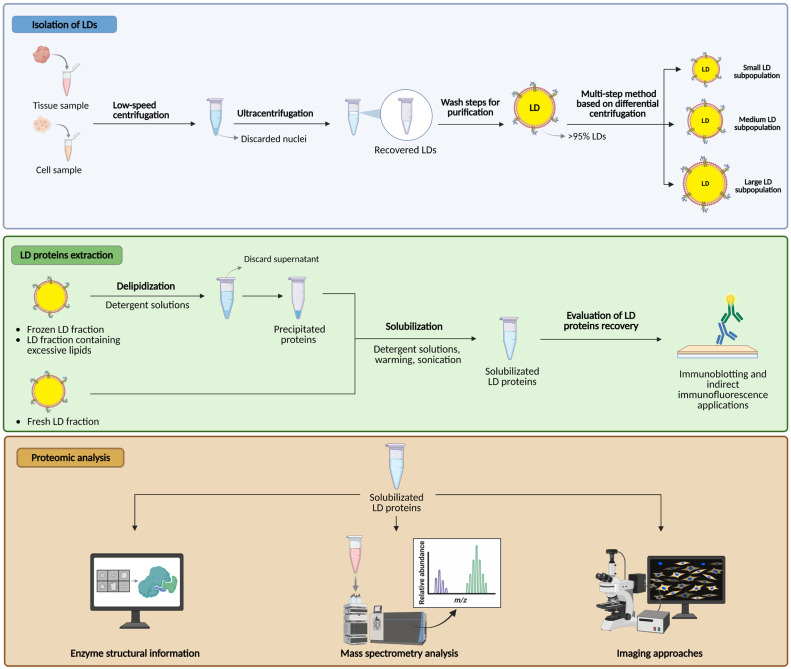
Overview of the technologies for isolation, extraction, and proteomic analysis of LDs. LD, lipid droplet. Created in Biorender. G.G.P. (2025) https://app.biorender.com (accessed on 20 October 2025).

A schematic representation of methodologies used to isolate, process, and analyze the proteome of LDs is reported in Figure 2.

The LD proteome in tissues and cell types can be mapped using a variety of methods and tools, such as enzyme structural information systems, mass spectrometry (MS) for both proteomics and lipidomics measurements [99,100], and imaging approaches [101]. Significant technological advances in MS, in combination with optimized purification and enrichment techniques, can provide the in-depth analysis of the LD proteome, enabling scientists to achieve higher analyte sensitivity and accuracy. Proteomic and lipidomic analyses based on MS are powerful strategies to identify and quantify changes in the abundance of neutral lipids and phospholipid species in LDs or LD-associated proteins in picomolar or nanomolar concentrations. In addition, high-resolution imaging techniques can be useful on specific cell types to uncover more information, for example, on the progression and regression of the atherosclerotic plaque [98].

Over the years, the proteome of LDs has been extensively characterized by proteomics in LD-enriched buoyant fractions isolated from many species and cell types [102,103,104,105,106]. Several protocols for isolating LDs by discontinuous density gradient centrifugation have been described [107]. Due to relatively low protein content and neutral lipid core, LDs are more buoyant than other cellular compartments and can be rapidly isolated by low-speed centrifugation. The buoyant density of LDs is <1 g/cm^3^. Briefly, the basic protocol involves the removal of nuclei by low-speed centrifugation and the adjustment of the density of the post-nuclear supernatant with sucrose before flotation of the LDs using a single discontinuous density gradient [107]. Generally, two low-speed centrifugation steps and a single ultracentrifugation step allow for collecting >95% of the LDs from a cell lysate.

The optimization of the centrifugation conditions is crucial and closely related to the size of the LDs in the starting sample. In general, a speed of 10,000× *g* for one hour is required for LDs to float well, but the speed of ultracentrifugation can be modified and adapted according to the LD size of interest [108]. Too high speed and duration of centrifugation may damage large LDs and can also detach proteins from LDs, while too low speed may result in a loss of small LDs.

The traditional protocol for LD isolation contains several washing steps after ultracentrifugation, but sometimes very small LDs can be lost during both these two steps, and supersized LDs can be impaired during homogenization and ultracentrifugation [108]. However, many wash steps are mandatory to improve LD purity before their collection.

Some precautions should be considered during the optimization of the isolation LD protocol. The isolation of LDs by density gradient centrifugation is quite a simple procedure, but the most critical step in the isolation of these droplets is the optimization of a reliable method of cell disruption, maintaining the integrity of LDs. During tissue homogenization, all samples should be fresh, because freezing can damage the LD structure. Gentle cell disruption is essential to preserve LD structure and obtain better recovery, and it is possible by using, for example, a hand-operated homogenizer with a teflon pestle or nitrogen decompression from a pressurized vessel, which are both effective ways to homogenize cells and tissues, releasing intact organelles [109]. The type of homogenizer, as well as the applied pressure and time, are key factors examined to obtain intact LDs during cell disruption and differ from sample to sample [108]. The use of hypotonic solutions and weak electrolytes helps to keep LDs intact and reduce their aggregation. In addition, to avoid protein degradation, it is also important to maintain the sample cold and to use protease inhibitors.

To obtain an efficient LD isolation, it is recommended to use small-diameter tubes, because the LD layer can spread and be easily disrupted in tubes with a wider volume during sample collection [107]. Moreover, a centrifugation using swinging bucket rotors allows the LDs to keep floating compactly at the top of the tube, which appears as a milky layer. In the event of poor isolation with a breakage and emulsification of the LDs, the layer at the top of the tube is transparent and oily, while the underlying bands appear very cloudy and white [107]. Particular care must be taken when collecting the floating droplet fraction to prevent a portion from staying attached to the sides of the tube and the surfaces of any pipetting device. By the use of pipetting devices or syringes with a wide-bore needle, it is possible to collect the LD fraction, but the Beckman tube slicer in combination with thin-walled polyallomer or polycarbonate tubes allows a better recovery in a minimal volume [107]. In addition, care should be taken not to collect the buffer below the LD layer, as it contains contaminants (e.g., cytosol proteins and membranes).

Usually, the recovery and purity of the LD fraction are verified by SDS-PAGE of solubilized proteins and immunoblotting, evaluating the immunoreactivity against known LD-associated proteins and specific intracellular membrane marker proteins [107]. LD fractions contain a low amount of protein, unlike the high lipid content that can interfere with the resolution of proteins by SDS-PAGE. When the LD fractions are fresh, their membrane proteins can be solubilized using detergent solutions with warming and sonication of the sample. Instead, frozen LD fractions or fractions containing excessive lipids are usually delipidated using solvents (e.g., acetone), and the precipitated proteins solubilized in concentrated detergent solutions with warming and sonication [107,108]. Plin1 and Plin2 are useful markers of LDs in immunoblotting and indirect immunofluorescence applications; in particular, Plin2 is a valid marker for the evaluation of LD recovery in most types of cells, while Plin1 is a good marker for LDs isolated from cultured adipocytes or adipocytes in tissue samples.

The proteomic analysis of LDs is made difficult by the heterogeneity of LDs, including differences in LD size, organelle association, and lipid composition. Many false positives have been found due to contamination with co-fractionating organelles or membrane remnants, including the ER, mitochondria, peroxisomes, and endosomes.

The close association of LDs with membrane-bound cellular organelles makes it difficult to purify them homogeneously and accurately identify their proteome [110]. Indeed, LDs exist in proximity to other organelles and often form dynamic connections with them, creating functional interactions that promote lipid exchange and regulate organelle biogenesis and turnover. Therefore, it is challenging to define which proteins are truly localized to the LD surface rather than proteins transiently associated with LDs due to this inter-organelle crosstalk. Due to the difficulty in identifying the true LD proteome, there is a tendency to overestimate the LD proteome and misrepresent its function and regulation. The development of methods able to differentiate the true LD proteome could provide a more accurate and comprehensive profile excluding common contaminants.

Anyway, since membrane contact sites between LDs and other organelles are implicated in important biological processes in different metabolic and pathophysiological contexts, their study has also gained significant prominence in the research field, leading to the identification of LD protein tethers and regulatory factors [81].

Therefore, it is not only important to study the LD-associated proteins but also to better understand the interactions between LDs and other organelles, because they play pivotal roles in maintaining cellular energy homeostasis and managing oxidative stress.

Many years ago, a study by Ding et al. [111] reported the proteomic analysis of isolated LDs of white adipose tissue from C57BL/6 mice, and a total of 193 proteins was identified by liquid chromatography (LC)-MS/MS analysis, including proteins belonging to the Plin family, lipid metabolism, mitochondria, ER, membrane traffic, cytoskeleton, and cell signaling. Although the authors have washed the LDs several times to remove potential contaminants, proteins present in various intracellular organelles were also reported in this proteome, confirming that LDs are frequently attached to the ER and mitochondria.

In the cardiovascular field, as an example, the high number of mitochondrial proteins in cardiac LD fractions could be a true biological phenomenon, which demonstrates the dynamic interaction between LDs and mitochondria essential for cellular processes, but it could also possibly be caused by purification artifacts, including PDMs, that are difficult to separate from LDs during purification. Indeed, the LD fraction can still contain these contaminating organelles even after high-speed centrifugation, leading to the inclusion of their proteins in the isolated fraction.

Atherosclerosis and heart failure are significantly affected by dysfunctional interactions between LDs and mitochondria, due to alterations in LD dynamics and mitochondrial oxidative capacity with an excessive lipid accumulation [112]. Therefore, the exploration of LD and mitochondria interactions could allow us to understand the precise molecular mechanisms governing these interactions in a disease context, paving the way for innovative approaches to disease treatment and prevention.

However, since different cellular membranes may adhere to the LDs, not only mitochondria, several methods to obtain a highly purified LD fraction with minimal contaminating membranes have been developed.

In this regard, a multi-step method based on differential centrifugation was successfully developed some years ago to separate LDs by size into large, medium, and small subpopulations [113]. The distinct LD subpopulations isolated from various cells and mammalian tissues showed significant differences in their protein composition, membrane phospholipid content, degree of associated organelles, functional characteristics, and ability to recruit proteins. Interestingly, this method was rapid and easily applicable to several tissue and cell LD sources with minor adaptations. The MS analysis allowed the identification of proteins in all three LD fractions and proteins expressed only in a specific fraction. In addition, the fraction containing small LDs was free of ER and mitochondrial marker proteins, suggesting that small LDs were not associated with these cellular organelles [113]. Therefore, the purification and separation of LDs by size and their association with other organelles are two important analytical steps for obtaining an accurate view of the proteome content.

To overcome the limitations associated with the highly reported abundance of ER and mitochondrial molecules in LD proteomes, another methodology based on a quantitative high-resolution MS-based proteomics strategy that employs protein correlation profiles (PCPs) was also successfully developed and applied in Drosophila cells by Krahmer et al., opening the way to the possibility of this approach to be used also in other cell types and tissues with high confidence [110]. Anyway, this approach failed to detect proteins that localize to multiple cellular compartments, thus requiring an advanced fractionation method to increase the purity of LD preparations.

To create high-confidence LD proteomic maps, a proximity-labeling proteomics approach that exploits an engineered ascorbate peroxidase (APEX2) for the selective fusion with LD-resident proteins was then developed, enabling the biotinylation of endogenous LD proteins in intact living cells and their following affinity purification for quantitative proteomic analysis by LC-MS [83,101,114]. This technology allows for maintaining organelle architecture and reducing post-lysis artifacts, thus generating high-confidence LD proteomic maps. LD-targeted APEX2 removes the abundant non-LD proteins commonly detected in LD proteomic investigations, which account for the vast majority of proteins found in buoyant fractions [83]. A ratiometric stable isotope labeling with amino acids in cell culture (SILAC) can also subtract cytosolic background labeling [115]; however, it does not completely distinguish the proteins that localize to more than one cellular compartment. Instead, a subcellular fractionation step is more useful to isolate biotinylated proteins on LDs from proteins present in the cytosol and other associated organelles, given that many known LD proteins localize to both LDs-ER (e.g., AUP1, GPAT4, and UBXD8) and LDs-cytosol (e.g., VCP, UBE2G2, and HSL) [101]. Therefore, proximity labeling allows the investigation of LD proteome dynamics and is a powerful tool for future studies on the remodeling of the LD proteome in response to metabolic signals in different disease contexts.

A Lipid Droplet Proteome database (https://www.dropletproteome.org/) was generated by the Olzmann laboratory and contains the MS/MS data from the study described above [83]. Similarly, a Lipid Droplet Knowledge Portal (https://lipiddroplet.org/) was created as an online platform that combines comprehensive datasets from systematic analyses of LD biology [116] and integrates gene expression, proteomics, and LD morphology data from a variety of cell types and species.

Technological advances in recent years have enabled a notable increase in studies on the LD proteome, and the role of its associated proteins has been successfully investigated in various pathologies related to lipid metabolic disorders.

It is well-known that lipid metabolic disorders are closely associated with progressive cardiac dysfunction and increased cardiovascular risk. In the heart and blood vessels, LDs accumulation and inflammation can significantly alter cardiac function, leading to lipid dysregulation, increased susceptibility to atherosclerotic plaque formation in the vessel wall, heart failure, and overall cardiomyopathies.

For these reasons, LDs and LD-associated proteins have attracted considerable attention in CVDs because of their high morbidity and mortality. The identification of key players in vascular and heart LD biology could improve our understanding of LD-linked diseases by clarifying the molecular and cellular processes that underlie lipid storage in cardiomyocytes and macrophage foam cells.

## 6. Lipid Droplets in the Cardiovascular Field

Despite the enormous knowledge on the role of LDs in adipose tissue and the liver, much still needs to be done to understand their involvement in heart and vascular tissue, the methods for studying them, and how LDs influence the development and progression of CVDs [98].

LDs play a key role in cardiac energy metabolism, maintaining proper amounts of FAs. Thus, an excessive accumulation of LDs is frequently associated with negative adverse biological effects on the heart and vessels, increasing the risk of unstable atherosclerotic plaques, hypertension, and hypertrophic cardiomyopathy. The abnormal cardiac lipid accumulation can change the redox state of the heart, leading to cardiomyopathy, a disease state that alters the myocardial function, causing heart failure and sometimes cardiac death [117].

Despite the significant progress made in elucidating the roles of LDs, there are still many details that need to be clarified about the LD proteome and how these proteins are involved in the development and progression of CVDs. LDs interact with various proteins to perform wider roles beyond lipid storage [98]. LD-associated proteins play a significant role in controlling the high turnover rate of LDs in the heart [117]. Therefore, increased or reduced levels of specific LD-associated proteins can induce myocardial dysfunction and contribute to the onset of CVDs.

Plins are certainly the most abundant proteins in LDs [96]. Lipid overload has a strong effect on Plin2, which is one of the most studied LD-associated proteins, and it is widely expressed in macrophages and atherosclerotic plaques. In addition, Plin5 is primarily expressed within oxidative tissues, such as the heart. Several studies from cell and animal experiments also showed that some other LD-associated proteins are involved in cardiac lipid regulation and cardiomyopathy development, such as ABHD5, of which levels are reduced in failing human hearts [118], or BSCL2, of which increased levels promote the massive cardiac lipid buildup [119,120], and ATGL, which instead helps the lipolysis process [117]. In particular, ATGL is mainly expressed in the adipose tissue and the breast, but it is also detected in cardiac tissue at the protein level [121]. ATGL knockout mice showed an accumulation of large amounts of TAGs in the heart with hypertrophic cardiomyopathy and interstitial fibrosis, causing cardiac dysfunction and heart failure or cardiac death [122]. Instead, cardiac-specific ATGL overexpression protected both diabetic and diet-induced obese mice from the development of cardiac steatosis and lipotoxic cardiomyopathy [123,124]. In addition, myocardial ATGL overexpression shielded against pressure overload-induced systolic cardiac dysfunction, altering the myocardial energy metabolism and improving heart function [125]. Similarly, cardiac-specific upregulation of ATGL ameliorated cardiac injury following serious burn challenge, alleviating cardiac lipid accumulation, thus demonstrating its therapeutic potential [126].

ATGL interacts with many other LD-associated proteins to exert several biological effects, such as Plin5 and ABHD5 with opposite dynamics. The interaction between ATGL and ABHD5 promotes lipolysis, while the binding with Plin5 recruits ATGL to the LD surface [127], causing a reduction in lipolysis in cardiomyocytes. In contrast, Plin2 is not directly connected to ATGL, but it is involved in reducing its connection with LDs, resulting in a decrease in TAG turnover [128]. It has also been observed that a deletion of BSCL2 increased ATGL protein stability and expression, together with a significant cardiac lipid remodeling [129].

Anyway, these results need to be further investigated and confirmed on human cardiomyopathies to convert these assumptions into practical applications.

Cardiovascular diseases are pathologies associated with the development of atherosclerosis, and LD-associated proteins have been reported to be linked to the development and progression of this chronic inflammatory process. Since foam cell formation is the central hallmark of atherosclerotic lesions, significant efforts have been made to explain the mechanisms underlying the abnormal accumulation of cytosolic LDs in foam cells and the role of LD-associated proteins in cholesterol efflux from macrophages [130]. Aiming to discover therapeutic strategies that target foam cells and prevent the progression of atherosclerosis, the study of the LD proteome is crucial to identifying novel targets of interventions. It is well-established that proteins from the cell death-inducing DFF45-like effector (CIDE) family, including CIDEB and CIDEC, are LD-associated proteins that are overexpressed during the foam cell formation in the atherosclerotic process [131]. These CIDE proteins play a significant role in regulating lipid metabolism and the formation of these lipid-filled foam cells. Similarly, Plins have a pivotal role in the atherosclerotic lesion formation and advancement [96]. Macrophage and atherosclerotic lesions also have a high expression of LD-associated hydrolase (LDAH) [130]. Numerous studies on the genetic modulation of these LD-associated proteins in mice have confirmed that they may be a promising new target to reduce atherogenesis by stopping foam cell formation.

Recently, a comprehensive quantitative proteomic and lipidomic analysis of human plaque-derived LDs has been performed to clarify the role of LDs in atherosclerosis and identify novel targets that are responsible for plaque pathology [132]. LDs have been isolated from different types of atherosclerotic plaques, classified as lipid-rich (LIP), mixed (MIX), and calcified (Ca). The study showed that the main proteins in the plaque LD proteome are secretory proteins, followed by cytoskeletal proteins and cytoskeletal regulatory proteins. In addition, LD-associated proteins correlate with the classification of atherosclerotic plaque better than the global tissue proteome, representing excellent indicators of proteome changes that occur with plaque progression. The LIP plaques, also known as “soft plaques”, contain more LD-resident proteins, such as Plin2 and acyl-CoA synthetase long chain family member 3, indicating that the lipid handling in these earlier-stage lesions is more active. As plaques progress and become calcified, the LDs transform from metabolically active organelles into inactive and fibrotic structures, structurally dominated by cytoskeletal proteins and cytoskeletal regulatory proteins.

Alterations in LD protein dynamics can lead to endothelial dysfunction. Indeed, abnormal accumulation of LDs and their dysregulated protein system, especially during atherosclerosis and inflammation, can lead to hypertension, impact endothelial function, and contribute to the progression of vascular disease [133]. In addition, atherosclerosis progression is accelerated by vascular smooth muscle cell senescence, due to lipid-mediated mitochondrial dysfunction, oxidative damage, and release of inflammatory factors [134].

In hyperlipidemic conditions, LDs accumulate in arterial walls, contributing to atherosclerosis, but also in the myocardial tissue, generating cardiac steatosis that is associated with the most well-known cardiovascular risk factors, such as obesity, diabetes mellitus, and hypertension, and frequently followed by heart failure [98]. Therefore, lipid metabolic disorders are closely associated with a progressive cardiac dysfunction [117].

Genetically modified mice with improved cardiomyocyte lipid uptake or impaired cardiac FA metabolism have been generated to investigate the impact of lipid overload on heart function. In cardiac lipotoxicity models, lipid accumulation causes cardiomyopathy, heart failure, and, in some cases, premature death [135,136]. Indeed, the reduced LD capacity promotes oxidative stress and cardiac functional decline. In other mouse models, transgenic overexpression of DGAT1 in the heart reduces levels of toxic lipids and improves heart function; thus, increased TG droplet storage induced by DGAT1 may serve a cytoprotective role in lipid overload states [137,138].

Clinical studies also showed higher intramyocardial lipid deposition in human patients with heart failure and diabetes with or without obesity, whose gene expression profiles were similar to the Zucker diabetic fat rodent model, an animal model of lipotoxicity, and associated with contractile dysfunction [139]. Therefore, the lower capacity of LDs to efficiently and safely store large amounts of FAs and the abnormal accumulation of triglycerides in the heart cause an increased mitochondrial FA β-oxidation with greater oxygen consumption, impairing cardiac function.

The study of signaling, metabolite trafficking, and protein–protein interaction between LDs and other organelles is also essential to clarify the physiological significance of LDs and the impact of these processes on CVDs.

## 7. Proteomic Applications in the Study of Lipid Droplets in CVDs

Current research is mainly focused on the study of LDs and the functions of their associated proteins in adipose tissue, liver, and macrophages, and little is known about their role in cardiac lipid regulation and association with cardiomyopathies [117].

LDs play a different role depending on the cell type, and their associated proteins cover a variety of specific functions, such as regulating cellular protein location, degradation, and mediating inflammatory signaling pathways [140,141]. Although LD proteomic studies have identified proteins involved in various roles, the functional significance of most LD proteins has not yet been discovered.

Proteomic characterization of LDs has been successfully performed on cancer cells [99,100], placental tissue [142], skeletal muscle [143], and liver [60,144,145], but much less is known about the proteome composition of LDs and its dynamics in the cardiovascular field.

In cardiomyopathy, the retention of neutral lipids in cardiac LDs is frequently observed. Therefore, studying the cardiac LD proteome contributes to clarifying the molecular mechanisms that link cardiac steatosis and myocardial dysfunction.

Here, we reviewed the current state of knowledge in the cardiovascular field of the LD proteome, providing an overview of the main studies on this topic (Table 2).

Aiming to understand the mechanisms that lead to myocardial dysfunction from a steatotic cardiac disease, the cardiac LD proteome was investigated in normal and dysfunctional Sprague Dawley rat hearts [146]. After the buoyant LD fraction isolation by sucrose gradient, an isobaric tags for relative or absolute quantitation (iTRAQ) method allowed the identification of 752 heart-associated LD proteins, among which more than 450 proteins were never previously associated with LD. In particular, 27 proteins were increased and 16 proteins were decreased in LDs from post-pressure overload-induced dysfunctional hearts in comparison to normal hearts. Among these altered LD-associated proteins, ATGL showed significantly reduced levels, and dysferlin increased, under cardiac dysfunction, thus suggesting a link between their significant variations in heart LD and myocardial dysfunction [146]. The majority of the identified proteins were mitochondrial proteins, confirming the metabolic relationship between LDs and mitochondria in cardiomyocytes, followed by proteins involved in DNA replication, transcription, and protein translation and modification, such as several histone proteins and chaperones. Notably, many of the proteins identified were not classified in the known categories based on sub-cellular distributions and functions, thus requiring further analysis [146].

Myocardial dysfunction is frequently related to the accumulation of cardiac LDs, and it is well-known that lipophagy has a key role in controlling lipid levels in numerous tissues, including the heart [147]. Selectively lipophagy to promote cholesterol efflux in foam cells could be a potential therapeutic strategy to treat atherosclerosis, and several proteins regulate these processes in macrophage foam cells to preserve cellular lipid homeostasis. Therefore, increasing lipophagy might help to reverse lipid buildup in atherosclerosis [148]. The study of lipophagy factors and their impact on LD degradation is crucial for understanding the causes and treatments of cardiometabolic diseases because impaired lipophagy is the underlying cause of various disorders, such as obesity, atherosclerosis, metabolic syndrome, and non-alcoholic fatty liver disease.

**Table 2:** Summary of proteomic studies performed on LDs in the cardiovascular field.

Disease	Study Model	Methods	Evidence	References
Atherosclerosis	Human differentiated THP-1 macrophages incubated with oxidized low-density lipoproteins	- Isolation and oxidation of LDL by sequential flotation ultracentrifugation - Induction and identification of foam cells - RNA isolation and microarray analysis - Western blotting analysis	- Stimulation of atherosclerosis development: increase in LD and foam cell formation, significant changes in LD-associated proteins (Plin and CIDE families), apolipoprotein family members, and PPAR family members	Li et al., 2010 [131]
Patients with atherosclerotic cardiovascular disease undergoing carotid artery surgery	Atherosclerotic plaques	- LD fraction isolation by density ultracentrifugation - LD separation by differential centrifugation - Lipid extraction and protein precipitation - Western blotting analysis - LC-MS/MS analysis	- Plin2, Plin3, and ACSL3 expression in the LD fraction - Multiple types of cytoskeletal proteins in plaque LDs: α-actin and β-actin, β-tubulin, vimentin, and keratin1 - Quantitative proteomic analysis on lipid-rich (LIP), mixed (MIX), and calcified (Ca): 2312 proteins in LD fractions shared across all groups (secretory, cytoskeletal, and cytoskeletal regulatory proteins) - LD-associated proteins correlated with plaque classification - Differential expression analysis (LIP vs. MIX/Ca): 444 identified proteins (62 upregulated and 382 downregulated)	Xu et al. 2025 [132]
Myocardial dysfunction from a steatotic cardiac disease	Dysfunctional Sprague Dawley rat hearts vs. normal rat hearts	- LD fraction isolation by sucrose gradient - iTRAQ quantitative LC-MS/MS analysis	752 identified proteins: - 27 upregulated (e.g., dysferlin) - 16 downregulated (e.g., ATGL)	Li et al., 2016 [146]
Myocardial disfunction from a cardiac LD accumulation	Differentiated human THP-1 macrophages incubated with agLDL and cultured under basal conditions or upon autophagy inhibition	- LD fraction isolation by density ultracentrifugation - LC-ESI-MS/MS	1265 identified proteins: Dysregulated levels of many lipophagy factors: - Structural proteins (e.g., Plin2) - HMGB1, HGMB2 - Lysosomal proteins - Rab proteins - Stress proteins - Ubiquitination factors - Selective autophagy receptors	Robichaud et al., 2021 [149]
Obesity-induced cardiomyopathy	Hearts of adult high-fat diet-feeding mice with obesity	- Western blotting analysis and immunofluorescence - Co-IP assay - LC-MS/MS analysis	RTN3 overexpression: - Increased myocardial LD content - Reduced left ventricular ejection fraction, left ventricular fractional shortening and E/A ratio - Increased level of FABP5	Guo et al., 2024 [150]
	Multiple models with both cardiomyocyte-specific RTN3 knockout or RTN3-overexpressing mice	- Western blotting analysis - IHC	RTN3 overexpression: - Intramyocardial lipid buildup and cardiac failure	
	Right atrial tissues from obese human patients	- Oil Red O staining - Western blotting analysis - IHC - Co-IP assay	- Increased LD biogenesis - Higher intramyocardial lipid overload and cardiac dysfunction - Increased level of RTN3 - Increased level of RTN3-FABP5 interaction	
	Lipotoxic hearts of obese C57BL mice	- LD fraction isolation by sucrose gradient - Co-IP assay - LC-MS/MS analysis	- Reduced Mfn2 levels - Inhibition of mitochondria-LD membrane contact sites - Maladaptive lipotoxicity	Hu et al., 2024 [151]
	Cardiac-specific Mfn2-KO mice	- Western blotting analysis - co-IP assay	- Increased Mfn2 acetylation and ubiquitylation - Reduced binding of Mfn2 with Hsc70 - Increased intramyocardial lipid accumulation	
	Cardiac tissue from human patients with obesity and non-obesity	- Western blotting analysis - Co-IP assay - Fluorescence imaging	- Extensive intramyocardial LD accumulation - Reduced Mfn2 levels - Increased Mfn2 acetylation and ubiquitylation - Reduced binding of Mfn2 with Hsc70	
	Palmitate-treated human iPSC-derived cardiomyocytes			

agLDL, aggregated low-density lipoprotein; ATGL, adipose triglyceride lipase; CIDE, cell death-inducing DFF45-like effector; Co-IP, co-immunoprecipitation; ESI, electrospray ionization; FABP5, fatty acid binding protein 5; HMGB, high mobility group box; Hsc70, heat shock cognate protein 70; IHC, immunohistochemical staining; iPSC, induced pluripotent stem cell; iTRAQ, isobaric tags for relative or absolute quantitation; LC, liquid chromatography; LD, lipid droplets; Mfn2, mitochondrion-localized mitofusin 2; MS, mass spectrometry; Plin2, perilipin 2; PPAR, peroxisome proliferator-activated receptors; RTN3, reticulon 3.

Robichaud et al. studied for the first time the LD-associated proteins of the lipophagy machinery by MS in differentiated human THP-1 macrophages, which were previously incubated with aggregated low-density lipoprotein (agLDL) to promote LD and foam cell formation and cultured under basal conditions or upon autophagy inhibition by chloroquine [149]. Besides structural LD proteins (e.g., Plin2, Plin3, and vimentin), metabolic enzymes (acyl-CoA synthetase long chain family members 3–4), and neutral lipases, other LD-specific proteins have been found, and they are involved in lipid transport, vesicular transport, lysosomal function, ubiquitination (e.g., AUP1), and autophagy regulation (e.g., coronin 1A, LAMTOR5, death-associated protein, desmocollin 1, and scavenger receptor class B, member 2) [149]. Plin2 is the major structural protein of macrophage foam cell LDs and is significantly elevated compared to non-foamy macrophages. Plin2 protects LDs against autophagy-mediated degradation, while its AMPK-dependent phosphorylation or deletion promotes lipophagy. In this study, the expression of putative lipophagy factors was altered on macrophage foam cell LDs, thus contributing to atherosclerosis development. In addition, the knocking down by siRNA of the lipophagy candidate genes resulted in a significant reduction in cholesterol efflux from murine macrophage foam cells, indicating the role of these proteins in lipophagy-mediated LD catabolism. Therefore, several molecular chaperones, high mobility group box 1 and 2, lysosomal proteins, Rab proteins, stress proteins, ubiquitination factors, and selective autophagy receptors have been recognized as active regulators of lipophagy [149]. Several aggresome components, including AUP1, OPTN, HSPA5, VCP, and 14-3-3 proteins, were also identified on LDs, and both single and aggregates of LDs were observed to be tagged for autophagic degradation in macrophage foam cells. In conclusion, since the expression levels of many lipophagy factors identified in this study are dysregulated in macrophages during atherosclerosis development, the impaired lipophagy pathway is supposed to contribute to atherogenesis. Therefore, the stimulation of the lipophagy process could be useful in the treatment of heart and other metabolic diseases.

Obesity significantly increases the risk of developing pathological conditions such as dyslipidemia, diabetes mellitus, CVDs, hypertension, and stroke [111], and all these cardiometabolic disorders are characterized by excessive or defective accumulation of LDs.

Recently, lipid overload-induced cardiac dysfunction was studied in the hearts of mice fed with a high-fat diet to induce obesity, which showed a significant increase in cardiac LDs and reticulon 3 (RTN3) expression, associated with a myocardial function impairment [150]. RTN3 is widely expressed in various tissues, including the heart, and its overexpression contributes to the pathological lipid accumulation through the binding with fatty acid binding protein 5 (FABP5), which promotes the transport of FAs to ER and LD biogenesis. Western blotting analysis confirmed the significant cardiomyocyte-specific RTN3 overexpression in adult mice upon high-fat diet (HFD) feeding. In addition, correlation analysis revealed a positive link between RTN3 levels and myocardial LD content, but a negative correlation with cardiac function indices such as left ventricular ejection fraction, left ventricular fractional shortening, and E/A ratio. Therefore, the upregulation of RTN3 in obese mice’s hearts leads to abnormal lipid accumulation and impaired cardiac performance [150]. Multiple models with both cardiomyocyte-specific RTN3 knockout and RTN3-overexpressing mice also demonstrated that the overexpression of RTN3 generated intramyocardial lipid buildup and cardiac failure in mice fed with a normal diet, thus reproducing the phenotype caused by a high-fat diet. Co-immunoprecipitation coupled with LC-MS/MS analysis allowed the identification of FABP5 as the main interactor of RTN3, with higher expression levels in the hearts and isolated adult cardiomyocytes of high-fat diet-feeding mice [150]. RTN3-mediated lipid accumulation was also studied in right atrial tissues obtained from obese human patients, and the protein levels of RTN3 and RTN3-FABP5 interaction were significantly enhanced in their hearts, accelerating the LD biogenesis in cardiomyocytes and leading to excess intramyocardial lipid overload together with cardiac dysfunction [150]. In conclusion, this study shows clinical potentialities and suggests RTN3 modulation as a possible therapeutic strategy for treating cardiac dysfunction in obese patients.

The excessive intramyocardial lipid accumulation in individuals with obesity leads to impaired myocardial function, but the endogenous pathway causing the transition from adaptive lipid utilization in healthy subjects to maladaptive lipotoxicity in obesity is still poorly understood. In a healthy heart, FAs released during lipolysis from LDs are delivered to mitochondria for ATP generation via β-oxidation. Therefore, the close association between LDs and mitochondria plays a crucial role in cardiac metabolic regulation. Anyway, the specific molecular mechanisms connecting LDs with mitochondria in cardiomyocytes and the physiological role of this tight connection have not yet been fully clarified.

Recently, it has been shown, for the first time, that mitochondrion-localized mitofusin2 (Mfn2) directly interacts with LD-localized heat shock cognate protein 70 (Hsc70), generating a complex that promotes FA transfer from LDs to the mitochondria for oxidation in cardiomyocytes [151]. In a lipid overload condition, Mfn2 expression is initially increased at both the transcriptional and protein levels following higher lipid supply, with an adaptive lipid utilization in cardiomyocytes. However, when lipid accumulation is excessive and prolonged, a reduction in Mfn2 occurs, inhibiting mitochondria-LD membrane contact sites, hindering FA oxidation, and activating a maladaptive lipotoxicity. Reduced LD-mitochondria connectivity associated with diminished Mfn2 levels was observed in lipotoxic hearts of obese C57BL mice [151], from which LDs were successfully isolated and purified from the other organelles. LC-MS/MS analysis was performed to identify LD proteins and proteins interacting with Mfn2 in the hearts of mice. The co-immunoprecipitation (co-IP) assays of the mouse heart lysates confirmed that Mfn2 interacted with Hsc70, whose levels were increased in LDs and localized on the LD surface. In addition, cardiac-specific knockout of Mfn2 in mice decreased mitochondria-LD membrane contact sites and increased intramyocardial lipid accumulation, contributing to lipotoxicity and consequent cardiac dysfunction. To validate the translational potential of these experimental data, cardiac tissue specimens from human patients with obesity and non-obese age-matched controls were collected, and higher intramyocardial LD levels were observed in obese patients than in controls, together with reduced Mfn2 levels [151]. Moreover, the increase in Mfn2 acetylation and ubiquitylation led to a decreased binding of Mfn2 with Hsc70 in the hearts of these patients, indicating that the majority of intramyocardial LDs were not connected to the mitochondria. Similarly, significantly decreased Mfn2 levels were observed also in palmitate-treated human-induced pluripotent stem cell-derived cardiomyocytes with associated extensive accumulation of LDs and severe HFD-induced cardiomyopathy [151]. In conclusion, a novel lipid overload-induced Mfn2 post-translational modification (PTM) pathway was proposed to be involved in the transition from adaptive lipid utilization to maladaptive lipotoxicity. Therefore, controlling mitochondria-LD membrane contact sites could be a potential therapeutic strategy in lipid overload-related cardiac dysfunctions.

## 8. Conclusions and Future Perspectives

The comprehensive proteomic analysis of LDs revealed the dynamic nature of their associated proteins, underscoring their multifaceted functions in lipid storage and metabolism, cellular stress responses, signaling, and organelle interaction. The functional characterization of these proteins is crucial for understanding the regulatory mechanisms that govern LD formation and turnover. In addition, the proteomic study of LDs provided a mapping of the associated molecules in different cell types or physiological conditions, as well as new insights into their involvement in several diseases such as obesity, liver disease, CVDs, and cancer.

The integration of emerging molecular profiling technologies with cardiovascular research offers unprecedented opportunities to unravel the complex biology of LDs in health and disease. The recent development of live-cell, isolation-free LD proteome mapping tools, such as LD-targeting chemical probes, enables high-resolution characterization of LD-associated proteins directly within their native microenvironment [152]. Applying these approaches to cardiovascular models and patient-derived tissues may reveal novel regulators of lipid storage, turnover, and signaling with direct relevance to atherosclerosis, myocardial lipid overload, and vascular inflammation.

Systematic profiling of PTMs represents a further tool for monitoring LD protein networks, which are regulated via several PTMs such as phosphorylation, ubiquitylation, acetylation, and palmitoylation [153]. PTMs of the LD proteome can modify the functions, stability, localization, and interactions between these organelles, driving important signaling pathways. Understanding the complex architecture of these signaling networks and how they interact with each other would allow us to clarify the role of LD proteins as substrates or regulators.

In addition, the combination of proteomic data with lipidomics, transcriptomics, and metabolomics would enable multi-omics models to unveil the functional outcomes of LD proteome dynamics, defining the roles of LDs in metabolism, signaling, stress response, and disease pathogenesis. This systems-level approach may facilitate the identification of causal relationships between LD remodeling and pathological processes such as lipotoxic cardiomyopathy, endothelial dysfunction, and foam cell formation. In parallel, dissecting the molecular regulation of LD-organelle contact sites, particularly with mitochondria, ER, and lysosomes, will be critical for understanding how lipid utilization, energy metabolism, and proteostasis are perturbed in cardiovascular disease [153].

Single-cell and spatially resolved proteomics are another exciting and rapidly evolving research area for studying heterogeneity in LD protein composition and specific cell types or disease subpopulations (e.g., in cancer or steatosis). Moreover, spatial proteomics allows mapping where proteins are localized in LDs, which typically move and interact with other organelles. Spatial proteomic approaches enable the precise mapping of LD-associated proteins and their subcellular interactions, revealing how specific protein complexes coordinate fatty acid transfer and oxidation at organelle contact sites. These spatially resolved datasets, when integrated with transcriptomic, lipidomic, and metabolomic information, provide a multidimensional view of LD biology—linking protein localization with metabolic flux and functional outcomes.

The application of spatial proteomics and multi-omics integration is opening new avenues for understanding LDs as dynamic and multifunctional organelles. Rather than serving solely as lipid storage sites, LDs are now recognized as metabolically active hubs that interact with multiple cellular compartments, including mitochondria and the endoplasmic reticulum, to regulate lipid trafficking, energy metabolism, and stress responses [101]. In the cardiovascular context, such integrative analyses hold the potential to elucidate how alterations in LD composition, distribution, and inter-organelle communication contribute to metabolic remodeling, lipotoxicity, and energetic dysfunction in the heart and vasculature. Ultimately, combining spatial proteomics with other omics layers may uncover novel molecular signatures and therapeutic targets related to lipid storage and utilization, offering a systems-level understanding of LD dynamics in cardiovascular health and disease.

Therefore, expanding the current proteomic landscape of LDs would lead to the identification of novel proteins, paving the way for a deeper understanding of LD biology and their role in cellular homeostasis and disease. The identification of key regulatory proteins is a starting point for future research into their contribution to metabolic diseases and offers potential biomarkers or targets for intervention. Indeed, the wider knowledge of the LD proteome would provide valuable information for an early diagnosis and prognosis in patients affected by lipid-related disorders and for the improvement of therapeutic strategies.

## Figures and Tables

**Table 1:** Main characteristis of class I and class II LDs proteins. CCT, CTP:phosphocholine cytidylyltransferase; ER, endoplasmic reticulum; DGAT, diacylglycerol O-acyltransferase; GPAT, glycerol-3-phosphate acyltransferase; LD, lipid droplet; Plins, perilipins.

	Class I Proteins	Class II Proteins
Origin	ER	Cytosolic ribosomes
Recruitment mechanism to LD	Incorporated during LD biogenesis	Recruited after LD formation
Structure of proteins’ targeting regions	V-shaped α-helical domain	Amphipathic helices or short hydrophobic-rich sequences
Turnover	Probably require dedicated machinery to extract them from LD membranes after relocalization of the protein to the ER or by direct extraction and degradation at the LD	Displaced from the LD surface by protein crowding during lipolysis and could be degraded by the ubiquitin/proteasome system
Examples	GPAT4, DGAT2, and ACSL3	Plins and CCT

## Data Availability

No new data were created or analyzed in this study. Data sharing is not applicable to this article.
